# Investigating the Relationship between of Vascular Endothelial Growth Factor and HER-2neu in IHC Staining with Metastasis and Mortality in Patients with Osteosarcoma

**DOI:** 10.31557/APJCP.2020.21.10.3005

**Published:** 2020-10

**Authors:** Mozaffar Aznab, Meisam Khajevand Ahmady, Khodamrad Jamshidi, Seyed Hamid Madani, Sedigheh Khazaei, Tina Shoushtaryzadeh, Abolfazl Bagheri

**Affiliations:** 1 *Internal Medicine Department, Talaghani Hospital, Kermanshah University of Medical Sciences, Kermanshah, Iran. *; 2 *General Hospital, Kermanshah, Iran. *; 3 *Department of Orthopedic Surgery, Shafa Orthopedic Hospital, Iran University of Medical Sciences, Tehran, IR Iran. *; 4 *Department of Pathology, Molecular Pathology Research Center, School of Medicine, Kermanshah University of Medical Sciences, Kermanshah, Iran. *; 5 *Molecular Pathology Research Center, Imam Reza Hospital, Kermanshah University of Medical Sciences, Kermanshah, Iran. *; 6 *Oncopathology Research Center and Hasheminejad Clinical Research Developing Center (HCRDC), Iran University of Medical Sciences (IUMS), Tehran, Iran. *

**Keywords:** Osteosarcoma, VEGF, HER 2 neu, metastasis, chemotherapy, Target therapy

## Abstract

**Background:**

The expression of HER-2neu and vascular endothelial growth factor (VEGF) in patients with osteosarcoma may determine the response to treatment. These two factors are likely to be effective in cancer progression. This study aimed at investigating the prevalence of these two factors in the pathological samples.

**Methods:**

Pathological samples of patients with osteosarcoma collected at a cancer surgery center between 2017 and 2018 were evaluated, of which 37 samples were included. The samples were evaluated using the IHC technique by two pathologists.

**Results:**

12 women and 25 men with an average age of 26.7 years were studied. 21 patients (56.8%) developed metastases from the beginning or during follow-up, whereas 16 patients (43.2%) have not yet developed metastases. Regarding HER-2neu, 21 patients (56.8%) scored 0, 9 patients (24.3%) scored 1, 3 patients (8.1%) scored +2, and 4 patients (10.8%) scored +3. The VEGF intensity scores of 0, 1+, 2+, +3, +4 and were found in 7 (18.9%), 2 (5.4%), 18 (48.6%), 8 (21.6%), and 2 (5.4%) patients, respectively. The results of the study did not show a significant relationship between age, gender, metastasis, and positive expression rates of HER-2neu and VEGF.

**Conclusion:**

The high expression of VEGF (75.7%) in the studied samples should be considered and further studies on this biomarker in cases with osteosarcoma are recommended from different aspects. To achieve validated results and prove the results of this study, similar studies with a larger sample size should be performed, and using targeted therapy for angiogenesis in large scale trials should be considered.

## Introduction

Osteosarcoma is a malignant high-grade bone tumor of osteoblastic origin and is the most common malignant tumor in young people (Marulanda et al., 2008). It is more prevalent during adolescence and old age (Mirabello et al., 2009). The initial peak incidence occurs in the first two decades of a person’s life, whereas the second incidence occurs after the fortieth decade of life and reaches its peak after the 6^th^ decade of life (Steliarova-Foucher et al., 2004; Stiller, 2007; Mirabello et al., 2009). The overall incidence of this tumor is reported to be 3-5 cases and 2-4 cases per 1 million populations in men and women, respectively (Mirabello et al., 2009). Osteosarcomas are more likely to involve long bones, including the distal femur, proximal tibia, and proximal humerus. It has been reported that from 1973 to 2004, the 5-year survival rate in individuals under 25 years of age was 61.6%, in cases aged 25-59 years was 58.7%, and in cases aged over 60 years was more than 24.2% (Mirabello et al., 2009). A Multi-disciplinary team is needed to treat osteosarcoma, in which the medical oncologist and orthopedist play a key role. Chemotherapy regimens used to treat osteosarcoma include a high-dose Methotrexate, Adriamycin and Cisplatin therapy regimen (Isakoff et al., 2015) as well as a chemotherapy regimen used in a study by Aznab and Hematti, (2017). Combination chemotherapy regimens are associated with a high risk of complications and a complete recovery is likely to achieve in only 30 to 60% of cases, whereas the recurrence is highly expected. The most important cause of death in these patients is pulmonary metastasis (Campanacci,1999). In cases of systemic recurrence, there is no appropriate chemotherapy for this disease, and the response to treatment is poor; therefore, it is necessary to change the therapeutic strategy in treating this disease in both primary cases, to reduce recurrence and in case of systemic recurrence. Efforts have been made to find low-risk treatments. Targeted therapy has been shown to be effective in treating many cancers. Therefore, it is important to find a target for targeted therapy to treat osteosarcoma. The vascular endothelial growth factor (VEGF) and HER-2neu are the factors that have been widely considered over the past two decades, which have resulted in acceptable outcomes, such as increased survival (Isakoff et al., 2015; Scotlandi et al., 2005). VEGF is a key regulator of angiogenesis (Przybylski, 2009). It has shown that the formation of the vascular system is associated with tumor metastatic activity. HER-2neu plays an important role in cell growth and differentiation. HER-2neu receptor expression is 10 to 100 times more in cancer cells than normal cells. Immunohistochemical (IHC) methods can be used to analyze the expression of HER2 and VEGF in tumor tissue samples. This study aimed at investigating the prevalence of these two factors in patients with osteosarcoma.

## Materials and Methods

This study was conducted on all patients referred to the specialized cancer surgery hospital in Tehran in 2016-2017 diagnosed with osteosarcoma, of whom 37 pathological samples in the initial evaluation by a qualified pathologist were considered for immunohistochemical (IHC) staining. IHC staining of VEGF and HER-2neu was performed. To evaluate the color of these two markers, the IHC scoring system is used. The staining intensity of VEGF was as follows: it was scored 0 for no staining. Regarding staining intensities, 1-25%, 26-50%, 51-75%, and over 75%, were scored 1 +, 2 +, 3 +, and 4 +, respectively. HER2 staining score was as follows: no staining (0); weak or incomplete membrane staining in less than 10% of cells(1+); complete staining that is intense and within less than or equal to 10% of the invasive tumor cells (2+) or in less than 30% of the cells; uniform, intense membrane staining (3+) in more than 30% of cells. This assessment was performed by a pathologist, and the results were confirmed by another pathologist and recorded in the relevant checklist. The medical records of these patients were not found and the type of treatment was unknown. Only information, such as age, sex, metastasis, and being alive or dead was obtained based on information after making a phone call with these patients.

## Results

12 women and 25 men with an average age of 26.7 years were studied. Twenty patients (54.1%) are alive and 17(45.9%) have died.Ten (27%), 12 (32.4%), 9 (24.3%), and 5 (13.5%) patients were in the age groups of 10-19, 20-29, 30-39, and 40-49 years, respectively. In another age classification, 10 patients (27%) were less than 20 years old. 12 patients (32.4%) were in the decade (20-29) years. Patients (16.2%) were over 40 years of age.

Regarding HER-2neu, 21 patients (56.8%) scored 0, 9 patients (24.3%) scored 1, 3 patients (8.1%) scored +2, and 4 patients (10.8%) scored +3.In terms of the intensity of VEGF staining, the following results were obtained. The VEGF intensity scores of 0, 1+, 2+, +3, +4 and were found in 7 (18.9%), 2 (5.4%), 18 (48.6%), 8 (21.6%), and 2 (5.4%) patients, respectively.21 patients (56.8%) developed metastases from the beginning or during follow-up, whereas 16 patients (43.2%) have not yet developed metastases. The relationship between HER-2neu, metastasis, and mortality was assessed (Table 1). Also, the relationship between VEGF and the rate of metastasis and mortality was investigated. In 7 patients with VEGF intensity score of 0, 5 patients (31.3%) showed no metastasis, whereas 2 patients (9.5%) showed metastasis. In 2 patients with VEGF intensity score of 1+, both patients (9.5%) showed metastasis. In 18 patients with an intensity score of 2+, seven patients (43.8%) showed no metastasis, and 11 patients (52.4%) showed metastasis. In 8 patients with VEGF intensity score of 3+, two patients (12.5%) showed no metastasis, and 6 patients (28.6%) showed metastasis. In 2 patients with VEGF intensity score of 4+, both patients (12.5%) showed no metastasis. The subjects were evaluated regarding the relationship between mortality and VEGF intensity score (Table 1). In brief, 18.9% of pathology samples were found with positive HER-2neu expression (2+, 3+), whereas 81.1% with negative expression (scores of 0 and +1), and 75.75% were found with VEGF overexpression (scores of 2 +, 3 +, 4 +3). Also, 24.3% of the samples were identified with low VEGF expression (scores of 0 and 1 +). In terms of mortality rate, in the female group (12 women), 7 patients were alive and 5 patients died, whereas in the male group (10 men), 10 patients were alive and 15 patients died, which this difference was not significant (P=0.295). In terms of survival rate, there was no significant statistical difference (0.062) in different age groups, such as less than 20 years, 20 - 29 years, 30 to 39 years, and ≥40 years. In terms of the relationship between HER-2neu, sex, and positive expression, in 12 female patients, 8 negative in 3 female patients were positive, whereas in 25 male patients, 18 patients were positive and 7 patients were negative, which was not statistically significant. There was no significant relationship between the prevalence of HER-2neu and gender (P-value = 0.062). There was also no significant relationship between the prevalence of VEGF and sex (P-value = 0.942). In this study, there was no significant relationship between the prevalence of HER-2neu and metastasis (P-value 0.349) and also between the prevalence of VEGF and metastasis (P-value 0.392). Statistically, there was no difference in the amount of metastasis in men or women (P-value 0.19). 

**Table 1:** The Relationship between HER2, VEGF, Metastasis and Mortality

	Score	N	Metastas+	Metastas-	Live	Death	*P*-value
Her2neu	0	21 (56.8%)	12 (57.1%)	9 (56.3%)	9 (52.9%)	12 (60%)	No significant
	1	9 (24.3%)	7 (33.3%)	2 (12.5%)	3 (17.6%)	9 (60%)	
	2	3 (8.1%)	0	3 (18.8%)	3 (17.6%)	0	
	3	4 (10.8%)	2 (9.5%)	2 (12.5%)	2 (11.8%)	2 (10%)	
VEGF	0	7 (18.9%)	2 (9.5%)	5 (31.3%)	5 (29.4%)	2 (10%)	No significant
	1	2 (5.5%)	2 (9.5%)	0	1 (5.9%)	1 (5%)	
	2	18 (48.6%)	11 (52.4%)	7 (43.8%)	7 (41.2%)	11 (55%)	
	3	8 (21.6%)	6 (28.6%)	2 (12.5%)	2 (11.8%)	6 (30%)	
	4	2 (5.4%)	0	2 (12.5%)	2 (11.8%)	0	

## Discussion

In the last two decades, many efforts have been made to identify prognostic factors in osteosarcoma, by which predict the potentially diverse biological nature of osteosarcoma and categorize patients to select radical surgery and appropriate treatment options and identify patients at risk for recurrence. These markers should be simple and practical in terms of use and applicability and also their results should be appropriate and understandable for interpretation and they should provide quick results; they should have also high specificity and sensitivity (Ritter and Bielack, 2009). Due to the age, at which this cancer is developed and its complications, such as amputation and metastasis to the lungs, decision-making for the correct diagnosis and classification of this disease is highly sensitive (DuBois and Demetri, 2007). HER-2neu and VEGF are markers that have been widely studied. Given the limited current histological evidence for osteosarcoma prognosis, these biomarkers can be used for the prognosis and treatment of osteosarcoma by proving the association between HER-2neu and VEGF expression and osteosarcoma (Zhou et al., 2003). Several studies have been done on HER-2neu expression in bone sarcomas, especially osteosarcoma, and an expression level of unusually 10 to 63% has reported for HER-2neu in osteosarcoma (Onda et al., 1996; Gorlick et al., 1999; Akatsuka et al., 2002). Nelson et al., (2014) showed that HER-2neu expression is observed in more than 40% of patients with osteosarcoma (scores of 2+ and 3+) and recommended conducting studies to investigate the role of drugs, such as trastuzumab in the treatment of patients with sarcomas, including osteosarcoma (Nelson, 2014). However, Kilpatrick et al., (2001) reported no patients with a staining score of 3+, and in cases with other staining scores (1+, 0, 2+), no correlation was found between HER-2nu expression and response to treatment or prognosis (Kilpatrick et al., 2001). In our present study, the HER-2neu positive expression was about 19% (scores of 2+ and 3+). In this study, there was no significant relationship between HER-2neu and gender, age, and metastasis, which is consistent with the study by Kelpartik et al., (2001) study. Becker et al., (2013) did not find a significant relationship between the HER-2neu marker and patient prognosis. In their study, only 11% of patients showed overexpression of HER-2neu (3+); however, this expression had an inverse relationship with lung metastasis. One of the first studies to investigate HER-2neu in osteosarcoma was conducted by Onda et al., in which 57.69% of the patients were found with HER-2neu staining score of 0, 7.69% had a score of +1, 19.2% had HER-2neu score of +2, and 15.3% were found with HER-2neu score of +3. In other words, about 68.3% of the patients had a negative score (0 and 1+), whereas 34.5% of patients had a positive score (2 +, 3 +) (Onda et al., 1996), which those who scored 2+ and 3 + showed a higher rate of metastasis and was not consistent with our study. In a study by Gorlick et al., (1999) 42.6% of the patients showed overexpression of HER-2neu and also had a higher rate of metastasis. These cases also had a lower survival rate, which is not consistent with our results. In Scotlandi et al., (2005) study, 32% of patients with osteosarcoma showed overexpression of HER-2neu with the prognostic value of event-free survival. In some studies, different HER-2neu measurement methods have been suggested as possible causes of differences in results.

In general, the differences in the various and sometimes contradictory reports can be explained by the following factors: Different techniques for measuring HER-2neu, differences in tissue processing, differences in anti-HER-2neu antibody, different interpretations of the outcomes, different treatment protocols for each patient, and sample size. These factors can vary in different studies, and a difference in these factors, even to a slight degree, different results can be expected. In the present study, the IHC method was used. VEGF has been widely considered. In Kaya et al. found that VEGF was positive in 63% of samples with a higher rate of metastasis. Also, relapse-free survival and overall survival were higher in patients with VEGF-positive tumors. In Kaya et al., (2000) study, positive cases (membrane staining and cytoplasmic staining) was over 30%, and there was a significant relationship between VEGF expression and poor outcome of osteosarcoma, which is almost consistent with our results in terms of the VEGF-positive cases, but it is not comparable in terms of prognosis due to the lack of patients’ medical records. Kaya et al., (2000) reported that the high expression of VEGF in patients with osteosarcoma before receiving treatment indicates a poor prognosis and a higher risk of pulmonary metastasis. Oda et al., (2006) studied osteosarcoma specimens and reported that patients with positive-VEGF tumors had a significantly poorer prognosis and these cases, were more likely to lung metastasis in the future (Ek et al.,2006). Ek et al., (2006) did not find a significant relationship between high VEGF expression and clinicopathological parameters, such as local recurrence, metastasis, and death in cases with osteosarcoma tumors. Also, there was not a significant correlation between VEGF staining intensity and in patients’ age. Kaya et al., (2000) and DuBois and Demetri, (2007) reported a relationship between positive VEGF expression with stage and grade of osteosarcoma tumors, as well as a correlation between positive VEGF expression and patient survival and lung metastasis. Lin et al., (2011) also showed that 64.3% of the patients had a high expression level of VEGF. In the present study, this rate was 75.7%, which indicates a high level of VEGF expression. Lin et al., (2011), showed a significant correlation between VEGF expression and metastasis with blood flow; however, in the present study, the relationship between VEGF and metastasis was not significant (P-value = 0.392). Lin et al., (2011) in a 2-year follow-up study showed that 66.7% of patients with positive-VEGF tumors developed metastasis, whereas, in patients with normal VEGF expression, only 35% of cases developed metastasis. The results of Lin et al., (2011) study showed no significant relationship between positive VEGF expression in pathological samples and age, sex, and tumor stage, which is consistent with the present study regarding the relationship between VEGF positive expression and age and sex. Becker et al., (2013) also found no significant relationship between VEGF and patient prognosis. The association between high VEGF expression and poor disease outcomes encourages researchers to use anti-angiogenesis drugs to improve the treatment of patients with malignant tumors (Yu et al., 2014). Sheikh et al., (2016) focused in detail on all targeted and molecular therapies to treat osteosarcoma and reported anti-VEGF drugs ineffective in the treatment of osteosarcoma alone; however, they stated that in the second phase of clinical trials to assess the combination of these drugs (Bevacizumab) with chemotherapy is conducted. They also reported that considering the HER-2neu expression rate of 40% in osteosarcoma patients, the results in terms of the relationship between this marker and patients’ poor prognosis, recurrence rate, and metastasis are contradictory. Therefore, they announced this relationship significant, whereas others consider it insignificant (Shaikh et al., 2016). Small sample size was one of the limitations of this study.

In Conclusion, according to the results of the present study, HER-2neu was not an important biomarker in osteosarcoma tumors (positive HER2 expression of 19%). On the other hand, the high expression of VEGF (75.5%) in the studied samples should be considered, and further study of this biomarker in the osteosarcoma samples regarding different aspects is recommended. Overall, the results of the present study did not show a significant relationship between age, gender, metastasis, positive HER-2neu expression, and positive VEGF expression. It is recommended that similar studies with a larger sample size be performed to obtain better results and further prove the results of this study. If possible, other biomarkers should be evaluated for osteosarcoma malignancy. If necessary, anti-angiogenesis and HER-2neu drugs in case of metastasis can be used.
